# Mapping of Photochemically-Derived Dityrosine across Fe-Bound N-Acetylated α-Synuclein

**DOI:** 10.3390/life10080124

**Published:** 2020-07-27

**Authors:** Alyson M. Curry, Ricardo D. Fernàndez, Talita D. Pagani, Dinendra L. Abeyawardhane, Morgan L. Trahan, Heather R. Lucas

**Affiliations:** Department of Chemistry, Virginia Commonwealth University, Richmond, VA 23284, USA; curryam@vcu.edu (A.M.C.); fernandezrd@vcu.edu (R.D.F.); paganit@vcu.edu (T.D.P.); abeyawardhadl@vcu.edu (D.L.A.); morgan.trahan@richmond.edu (M.L.T.)

**Keywords:** PICUP, dimerization, alpha-synuclein, photochemical crosslinking, iron, metals, Parkinson’s disease, intrinsically disordered proteins, dityrosine, protein-protein interactions, protein dynamics

## Abstract

Parkinson’s disease (PD) is the second most common neurological disease and belongs to a group of neurodegenerative disorders called synucleinopathies in which pathological aggregates of N-terminally acetylated α-synuclein (^NAc^α-Syn) accumulate in various regions of the brain. In PD, these ^NAc^α-Syn aggregates have been found to contain covalent dityrosine crosslinks, which can occur either intermolecularly or intramolecularly. Cerebral metal imbalance is also a hallmark of PD, warranting investigations into the effects of brain biometals on ^NAc^α-Syn. ^NAc^α-Syn is an intrinsically disordered protein, and metal-mediated conformational modifications of this structurally dynamic protein have been demonstrated to influence its propensity for dityrosine formation. In this study, a library of tyrosine-to-phenylalanine (Y-to-F) ^NAc^α-Syn constructs were designed in order to elucidate the nature and the precise residues involved in dityrosine crosslinking of Fe-bound ^NAc^α-Syn. The structural capacity of each mutant to form dityrosine crosslinks was assessed using Photo-Induced Cross-Linking of Unmodified Proteins (PICUP), demonstrating that coordination of either Fe^III^ or Fe^II^ to ^NAc^α-Syn inhibits dityrosine crosslinking among the C-terminal residues. We further demonstrate that Y39 is the main contributor to dityrosine formation of Fe-bound ^NAc^α-Syn, while Y125 is the main residue involved in dityrosine crosslinks in unmetalated ^NAc^α-Syn. Our results confirm that iron coordination has a global effect on ^NAc^α-Syn structure and reactivity.

## 1. Introduction

Synucleinopathies are a group of neurodegenerative disorders in which insoluble aggregates, made up predominantly of the neuronal protein α-synuclein (αSyn), accumulate in neurons, glial cells, and nerve fibers [1]. The toxicity of the αSyn aggregates manifests through many different biological pathways, such as abnormal mitochondrial activity, membrane damage, and impaired synaptic function among others [2,3]. Parkinson’s disease (PD) is perhaps the most notorious synucleinopathy, in which the progression of these abnormalities results in the death of dopaminergic neurons in the *substantia nigra*, leading to the distinctive motor symptoms associated with the disorder [4,5].

αSyn has long been considered an intrinsically disordered protein (IDP) because it is natively unfolded in its monomeric state [6,7,8]. It is constitutively acetylated at the N-terminus in humans (^NAc^αSyn), and this post-translational modification (PTM) has dramatic consequences on the metal binding characteristics and self-assembly mechanisms of this structurally dynamic protein [9,10,11,12]. The primary sequence of ^NAc^αSyn is partitioned into three distinct regions, each of which have distinct functions. The N-terminus (residues 1–60) has a series of imperfect repeats with a core consensus sequence [13]. The repeats help to form amphipathic helices that deter the formation of β-structures and help the protein to interact with lipid membranes. The succeeding region of ^NAc^αSyn (residues 61–95) is known as the Non Amyloid-β Component region (NAC) and holds hydrophobic residues that contribute to aggregate formation [14]. The C-terminus (residues 96–140) is an acidic region that helps to block fibril assembly and is postulated to help protect the protein from denaturation [15].

IDPs, including ^NAc^αSyn, are characterized by dynamic structural mobility, imparting them with the flexibility to access a variety of different conformations [6,16]. This suppleness can be envisioned as a shallow energy landscape with multiple local minima, in contrast to canonical ordered proteins which have a deep and discrete energy minimum representative of a single folded state [17]. Various factors can modulate the energy landscape of IDPs, making these biomolecules well-suited to participate in a myriad of biological interactions or processes depending on the physiological status of the overall system. External stimuli—metal binding, for example—can stabilize a particular structure that could have a downstream influence on a specific biological pathway. Metal dyshomeostasis has long been linked to PD [18,19,20], and victims of PD present with increased cerebral iron levels and diminished levels of copper in affected regions of the brain. Although the precise roles of brain metals on the function of ^NAc^αSyn have yet to be elucidated, we [10,21,22,23] and others [9,24,25] have demonstrated how prevalent brain biometals modulate the structural outcome of ^NAc^αSyn self-assembly pathways and influence both the potential and the extent of its aggregation.

**Figure 1:**
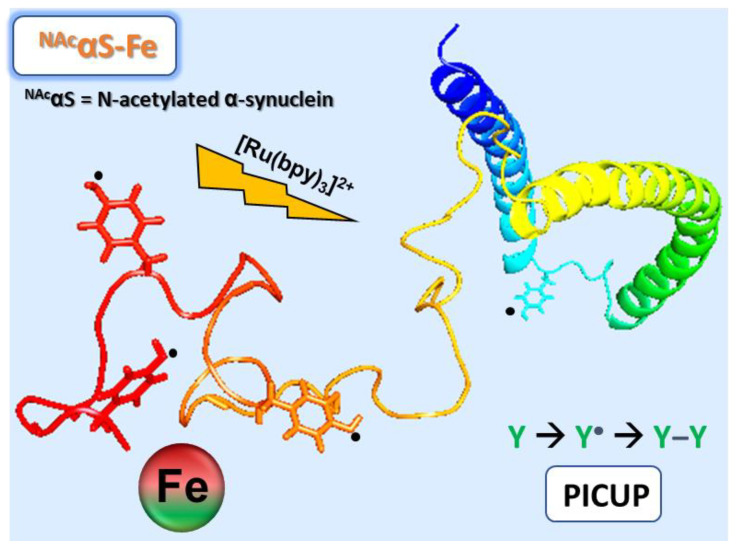
Cartoon representation of the photoinitiated crosslinking of ^NAc^αSyn. Each tyrosyl residue within the protein sequence is depicted, with Y39 shown in blue and the C-terminal tyrosines shown in orange/red (PDB 2KKW27).

Photoinitiated crosslinking of IDPs can be used to track the influence of a particular factor, shedding light on the biophysical ramifications and giving insight into potential biological consequences. To this end, we have employed Photo-Induced Cross-Linking of Unmodified Proteins (PICUP) to map the structural effects of iron binding to ^NAc^αSyn (Figure 1). The PICUP technique relies on a ruthenium photocatalyst to generate protein-based radicals via the one-electron oxidation of tyrosine, tryptophan, or cysteine, which can combine to generate covalent crosslinks within a protein or among proteins [26,27]. PICUP has been employed to stabilize metastable oligomeric populations of proteins with the aim of providing a ‘snapshot’ of oligomer assembly and size distribution [28]. Our group has recently reported its application for the evaluation of ^NAc^αSyn structural modifications that result from metal coordination, which provided insight on intramolecular interactions and global dynamics [23]. ^NAc^αSyn has a total of four tyrosines and no tryptophan or cysteine residues. Accordingly, only the Y39, Y125, Y133 and Y136 residues can participate in PICUP reactions, generating dityrosine crosslinks that can be measured by their intrinsic fluorescence [23]. Dityrosine crosslinks are common PTMs within post-mortem brain samples from PD victims [9,29]. They are considered indicators of oxidative stress as well as PD biomarkers, so understanding the extrinsic factors that promote or prevent ^NAc^αSyn structural configurations that make dityrosine crosslinks spatially accessible is warranted. In this article, we employ systematic tyrosine-to-phenylalanine ^NAc^αSyn mutations to examine the impact of iron binding on the global protein dynamics of ^NAc^αSyn. In our previous studies [10,21], dityrosine formation was cataloged after an extended aggregation process under fibrillation conditions, while in these studies the immediate impact of iron binding was monitored, so our data herein depict preferential interactions in an unrestricted dynamic system. Previous reports from our group that utilized the PICUP method examined the influence of metals on the wild type protein [23]; through the work described herein, we will be able to map the involvement of specific tyrosine residues in the crosslinking process.

## 2. Materials and Methods

### 2.1. Transformation of Expression Strains for ^NAc^αSyn Variants

Expression vectors for ^NAc^αSyn tyrosine-to-phenylalanine variants were constructed using the Q5 site-directed mutagenesis kit (New England BioLabs) as previously described for other ^NAc^αSyn variants [21]. Successful generation of each plasmid was confirmed by DNA sequencing (Eurofins Genomics). To generate the N-terminal acetylation that is a native post-translational modification of ^NAc^αSyn, each of the engineered ^NAc^αSyn expression plasmids underwent co-transformation with pNatB [30]. The pNatB vector has a pACYCduet-1 backbone and holds genes naa20 and naa25, which encode for the catalytic and auxiliary subunits, respectively, of the NatB acetylase complex from yeast. pNatB (pACYCduet-naa20-naa25) was a gift from Dan Mulvihill (Addgene plasmid # 53613; http://n2t.net/addgene: 53613; RRID: Addgene_53613). Both vectors were co-transformed into a chemically competent E. coli expression strain (BL21–DE3). The transformed cells were plated on 2YT agar plate media supplemented with 30 µg/mL of kanamycin and 25 µg/mL of chloramphenicol and incubated for 24 h at 37 °C. For each variant, a single bacterial colony was selected to inoculate 5 mL of sterilized 2YT media supplemented with respective antibiotics and incubated at 37 °C, 250 rpm for 16 h. Glycerol stocks were prepared from this culture and stored at −80 °C for future use of large expression cultures. This process was followed for each of the engineered plasmids.

### 2.2. Expression and Protein Purification

A 25 mL culture of 2YT media was supplemented with 30 µg/mL of kanamycin and 25 µg/mL of chloramphenicol, and inoculated from a glycerol stock of BL21-DE3 co-transformed with pNatB and an ^NAc^αSyn expression plasmid. The culture was incubated at 37 °C, 300 rpm for 16 h. A 1 L culture of 2YT media supplemented with antibiotics at 30 µg/mL of kanamycin and 25 µg/mL of chloramphenicol was inoculated with the seed culture and incubated at 37 °C, 250 rpm until its optical density (OD) at 600 nm reached an absorbance of 0.6. The culture was then induced with 1 mM of isopropyl β-D-q-thiogalactopyranoside (IPTG, GoldBio) and incubated with shaking for an additional 4 h for protein expression. Expressed cells were pelleted by centrifugation at 5000 rpm, 4 °C for 20 min. The supernatant was discarded, and the pellet stored at −20 °C for forthcoming purification.

The cell pellet was resuspended in 25 mL of lysis buffer (100 mM Tris buffer, 300 mM NaCl, 1 mM EDTA, pH 8.2) and 125 µL of 100 mM phenylmethylsulfonyl fluoride (PMSF, GoldBio) in order to reach the final concentration of 0.5 mM PMSF. Cells were lysed by sonication with a sonicator probe (QSonica Q500) at 10-sec-on/10-sec-off, amplitude 20% for 3 min 20 sec total time. The headspace of the container holding the lysed cells was sealed with parafilm and the empty volume was purged with a gentle stream of inert gas for 5 min. The container was capped and cells were boiled in a water bath for 10 to 15 min. Cell debris and other precipitate were pelleted by centrifugation at 13,000× *g*, 4 °C for 30 min. The collected supernatant underwent acid precipitation at pH 3.5–4.0 to further enrich the soluble fraction in ^NAc^αSyn variants. Precipitates were again pelleted by centrifugation at 13,000× *g*, 4 °C for 30 min. The supernatant was filtered with a 0.22 µm syringe filter and then collected in a 3.5 kDa membrane tube and dialyzed three times against 20 mM Tris and 0.5 mM PMSF, pH 8.5 at 4 °C for at least 90 min per round. The dialyzed sample underwent anionic exchange purification with an AktaStart FPLC system (GE Healthcare). The sample was loaded into a HiPrep DEAE FF 16/10 column (GE Healthcare) previously equilibrated in 20 mM Tris, pH 8.2. ^NAc^αSyn Y-F variants were purified under a linear gradient elution against 20 mM Tris, 1 M NaCl, pH 8.2 (B buffer) over the course of 18 column volumes (CV). The enriched ^NAc^αSyn Y-F variant fractions eluted between 14–18% of B buffer. Fractions corresponding to peaks were analyzed via UV-Visible (UV-Vis) spectroscopy. The collected fractions were again dialyzed three times under the aforementioned conditions at 4 °C for at least 90 min per round. The dialyzed sample was further purified on a HiScreen Q HP column (GE Healthcare) mirroring conditions to the DEAE column. Fractions containing pure ^NAc^αSyn Y-F variants eluted between 20–28% B buffer. The purity of the fractions was confirmed by SDS-PAGE. The pure fractions were collectively or individually quantified by UV-Vis (Hitachi U-3310). Each mutant’s theoretical molar absorptivity was computed using ExPASY pI/Mw tool [31], and the concentration of ^NAc^Quad was determined through a Bradford assay. All samples were flash frozen in liquid nitrogen and stored at −80 °C.

### 2.3. Photochemical Crosslinking of Unmodified Proteins (PICUP)

Flash frozen ^NAc^αSyn Y-F variant samples were thawed, and oligomers were removed from the sample by centrifugation using a 30 kDa nanosep filter (PALL). Samples were then salt exchanged against 20 mM MOPS, 100 mM NaCl, pH 7.00 using a PD-10 desalting column (GE). PICUP solutions (250 µL) were then standardized to 35 µM protein and supplemented with stoichiometric iron triflate salts. Tris(bipyridine) ruthenium(II) chloride (Ru(bpy)_3_Cl_2_) and ammonium persulfate (APS) were added at 10 µM and 200 µM, respectively. Control samples had the metal addition substituted in volume with buffer (20 mM MOPS, 100 mM NaCl, pH 7.00). The prepared samples were irradiated at 450 nm for 45 min in the absence of stray light. The irradiated samples were characterized using a fluorescence spectrophotometer (Hitachi F-4500). Dityrosine fluorescence emission was excited at 320 nm and recorded from 340 to 520 nm. Statistical significance of integrated peak areas were determined by one-way ANOVA at the following levels: * *p* < 0.05, ** *p* < 0.01, *** *p* < 0.001, **** *p* < 0.0005. Samples were further analyzed by gel electrophoresis. Aliquots (20 µL) were mixed with loading buffer 4X containing DTT 1X and separated on 10% SDS-PAGE gels stained with Coomassie blue R-250 and imaged with an Azure Biosystems imager. Samples that exhibited weak intermolecular crosslink bands were concentrated using 3 kDa nanosep filters for further analyses.

## 3. Results and Discussion

### 3.1. Photoinduced Dityrosine Formation of ^NAc^αSyn Variants in the Absence of Iron

**Figure 2:**
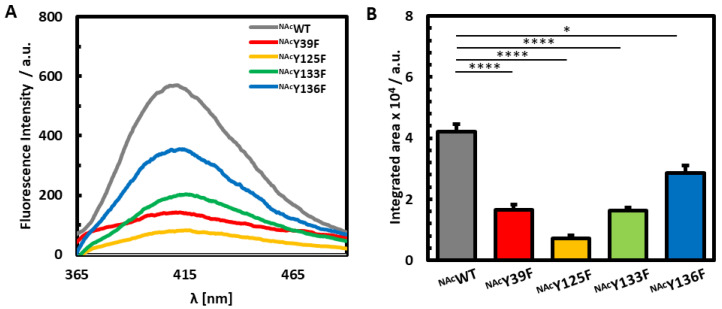
Fluorescence Analysis of No-Metal PICUP Samples. (**A**) Fluorescence spectra from no-metal added PICUP experiments performed on ^NAc^WT (grey), ^NAc^Y39F (red), ^NAc^Y125F (yellow), ^NAc^Y133F (green), and ^NAc^Y136F (blue). (**B**) Bar graphs representing the integrated area of their dityrosine signals.

**Figure A1:**
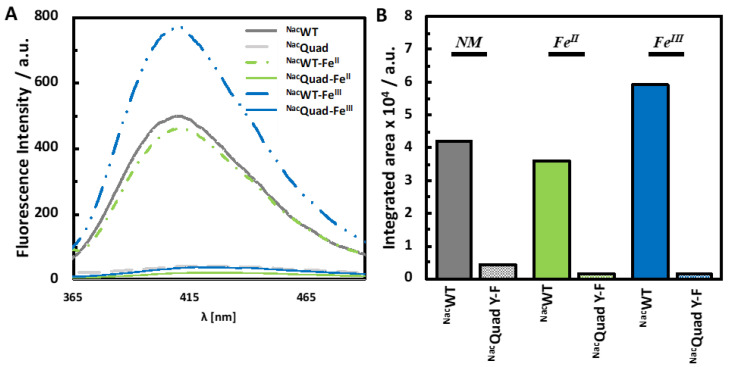
Fluorescence comparison between ^NAc^WT and ^NAc^Quad following PICUP. (**A**) Fluorescence spectra and (**B**) bar graphs representing the integrated area of no metal added (grey), Fe^II^-added (green), and Fe^III^-added (blue) of dityrosine signals on ^NAc^WT and ^NAc^Quad.

Tyrosine substituted ^NAc^αSyn variants were created through site-specific substitution of each tyrosine with phenylalanine in order to investigate their intrinsic differences in protein dynamics following the coordination of iron. N-terminal acetylation is achieved through coexpression with NatB acetylase, which targets proteins with starting M-D-X amino acid sequences [21,30]. To examine the impact of each individual tyrosine on the photochemical behavior of ^NAc^αSyn, PICUP studies were first performed in the absence of metal ions on ^NAc^WT, which retains the native sequence of ^NAc^αSyn, and each Y-to-F ^NAc^αSyn mutant (Figure 2A,B). A quadruple mutant (^NAc^Y39F/Y125F/Y133F/Y136F, or ^NAc^Quad) in which all four tyrosine residues were replaced with phenylalanine was also investigated. As expected, ^NAc^Quad had negligible fluorescence in the 365 nm–520 nm region in which dityrosine emission occurs (Figure A1), confirming that the quantum yield increases observed following photoillumination of ^NAc^WT and all tyrosine-containing ^NAc^αSyn variants result specifically from dityrosine formation.

Deletion of each individual tyrosine residue resulted in a significantly lower fluorescent dityrosine signal when compared to its native counterpart, with ^NAc^Y125F displaying the lowest emission. ^NAc^Y125F had a dityrosine signal reduced by 83% of the original ^NAc^WT intensity, indicating that Y125 is likely involved in the majority of the dityrosine crosslinks in the native form of the protein. Previous studies also found that Y125 is critical to the formation of αSyn dimers after nitrative stress [32]. Furthermore, human PD victims and drosophila PD models have demonstrated less phosphorylation at Y125, suggesting that a modification at this position could have a neuroprotective effect by inhibiting oligomerization [33]. All together these results are consistent with an integral function of Y125 in intracellular post-translational modifications.

In contrast, ^NAc^Y136F was demonstrated to contribute the least to dityrosine crosslinking, retaining 68% of the native dityrosine signal. Previous studies exposed the role of Y136 in the nucleation and extension of fibrils [34]. Stacking of aromatic residues through π–π interactions are known to contribute to fibrillation, but such an effect may be less apparent in dynamic fluctuations of the monomer. Our PICUP results indicate that Y136 is the least likely to participate in photoinitiated crosslinks among the four native tyrosine residues, suggesting that the inherent contortions of ^NAc^αSyn offer some protection from reactivity involving this residue, perhaps in order to maintain its accessibility to participate in non-covalent interactions.

^NAc^Y39F and ^NAc^Y133F each retained 39% of the original ^NAc^WT dityrosine crosslinking signal. We had previously reported that Y39 is a critical residue for the formation of intermolecular linkages [21]. The exact C-terminal tyrosine interactions could not be defined, yet it was concluded that the C-terminal residues are important for intramolecular crosslinking. Our current results may indicate a structural preference for Y39–Y133 intramolecular crosslinking events under photoirradiation of the dynamic monomeric form of ^NAc^αSyn due to their comparable fluorescence response under PICUP conditions. Alternatively, the similar reduction in fluorescence between ^NAc^Y39F and ^NAc^Y133F may represent a situation in which Y125 participates in dityrosine crosslinking with Y39 and Y133 indiscriminately, and that there is a larger energy barrier to crosslinking with Y136.

### 3.2. Photoinduced Dityrosine Formation of Fe-Bound ^NAc^αSyn Variants

**Figure 3:**
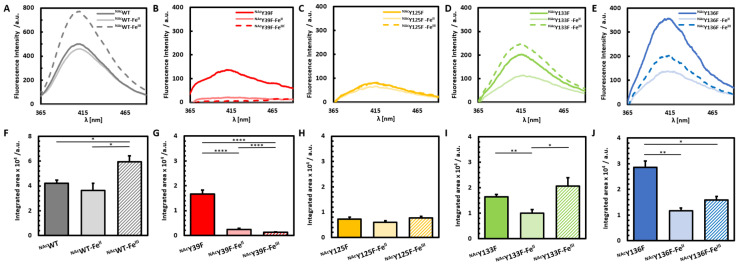
PICUP analysis of ^NAc^αSyn variants with and without iron added. Fluorescence spectra following PICUP reactions with no metal added (solid-dark), Fe^II^-added (solid-light), and Fe^III^-added (dashed/striped) of ^NAc^WT (**A**), ^NAc^Y39F (**B**) ^NAc^Y125F (**C**) ^NAc^Y133F (**D**) and ^NAc^Y136F (**E**). Bar graphs represent the integrated area of dityrosine emission for ^NAc^WT (**F**), ^NAc^Y39F (**G**) ^NAc^Y125F (**H**) ^NAc^Y133F (**I**) and ^NAc^Y136F (**J**).

Pathological concentrations of iron accumulate within the *substantia nigra* of PD afflicted patients [18]. To map the structural impact of iron coordination to ^NAc^αSyn, ^NAc^WT and each of the ^NAc^αSyn tyrosine mutants were subjected to stoichiometric ratios of iron in both the Fe^II^ and Fe^III^ redox states (Figure 3). Fe^II^ supplementation resulted in a moderate decrease in the overall fluorescence emission of dityrosine crosslinks within ^NAc^WT, while Fe^III^ supplementation enhanced overall dityrosine crosslinking in ^NAc^WT. Iron is thought to bind within the C-terminal region of ^NAc^αSyn [9,18,24], within close proximity to the three C-terminal tyrosine residues. Previously, we reported that Fe blocks Cu-induced photoinitiated dityrosine crosslinking in ^NAc^αSyn [23], and our current results further suggest that Fe^II^ also results in a relative decrease in dityrosine crosslinking compared to the unmetalated native protein. In contrast, Fe^III^ coordination to ^NAc^WT promotes a conformation of ^NAc^αSyn that facilitates photoinitiated dityrosine crosslinking.

Valuable insight can be gained by directly comparing the impact of Fe^III/II^ binding on photoinduced dityrosine crosslinking for each ^NAc^αSyn variant to its unmetalated counterpart. In the case of ^NAc^Y39F, this ^NAc^αSyn variant is only capable of C-terminal dityrosine crosslinking, as the remaining tyrosine residues (Y125, Y133, Y136) are all clustered within a short segment of the acidic C-terminal tail, which is also thought to house an Fe-binding site for ^NAc^αSyn [9,18,24]. Notably, iron supplementation of ^NAc^Y39F with either Fe^II^ or Fe^III^ nearly abolished all dityrosine formation, reducing the fluorescence signal down to ~5% of the native signal and indicating that Fe-binding effectively prevents dityrosine crosslinks from forming among any and all of the C-terminal tyrosine residues (Figure 3B). These results reinforce our previous conclusion that coordination of Fe^III/II^ to ^NAc^αSyn hinders intramolecular dityrosine crosslinking as well as our previous assertion that these intramolecular crosslinks derive from the C-terminal tyrosine residues [21,23].

When Y39 is not altered but one of the C-terminal tyrosine residues is instead removed, there is a less dramatic effect on photochemical crosslinking when Fe in either redox state is bound. This further supports a key role for Y39 in crosslinking when Fe is bound within the C-terminus. Our results are consistent with a model in which Fe-binding within the acidic C-terminal region of ^NAc^αSyn structurally prohibits the formation of intramolecular dityrosine crosslinks among Y125, Y133, and Y136 by stabilizing a structure in which these residues are not capable of ortho-ortho coupling with each other due to their relative spatial orientations. Our results further suggest that each of these C-terminal tyrosine residues is capable of participating in photoinitiated dityrosine crosslinks with Y39 that are either not disrupted or only minimally disrupted by iron binding.

The ^NAc^Y125F variant, which demonstrated the most dramatic reduction in photoinitiated dityrosine crosslinks in the absence of metal, showed no further reduction upon binding of either Fe^II^ or Fe^III^. These results suggest that, not only is Y125 responsible for most of the dityrosine crosslinking in unmetalated ^NAc^αSyn, but also that iron coordination has no further impact on reaction events in the absence of this residue. Altogether, our observations are also consistent with Y125 maintaining a critical role in promoting or preventing the toxic oligomerization of ^NAc^αSyn based on the type and extent of post-translational modifications at this site and/or the structural modulations controlled by this residue’s interactions.

Among the other C-terminally modified variants, Fe^II^ coordination resulted in a relative decrease in the amount of dityrosine crosslinking within both the Y133F and Y136F mutants. In contrast, Fe^III^ coordination resulted in a decrease in fluorescence following PICUP reactions with the Y136F variant, but resulted in a relative increase in studies involving the Y133F mutant. In a previous computational study on αSyn Y-to-A mutants, Y133A had a different folding pattern and was more flexible than the other tyrosine mutants [35], which would support the proposition that Y133 provides a steric obstacle to involvement in molecular interactions at this site.

**Figure 4:**
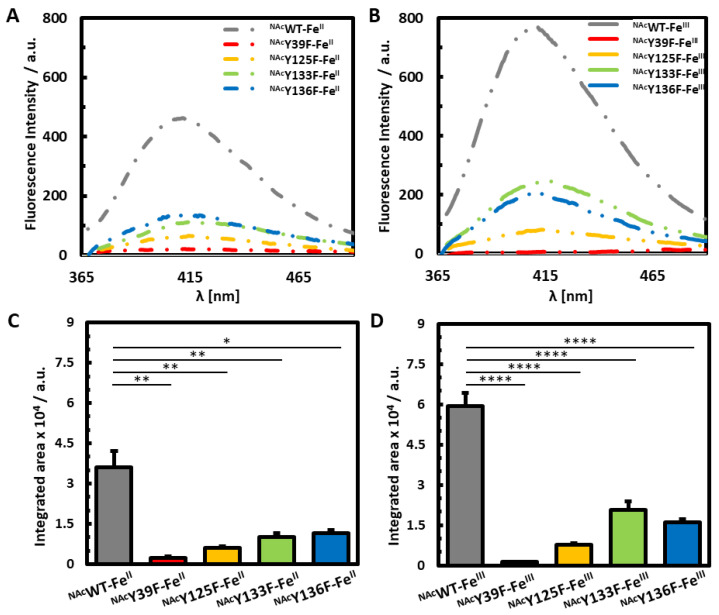
Fluorescence analysis of Fe-bound ^NAc^αSyn. Fluorescence spectra of (**A**) ^NAc^αSyn-Fe^II^ and (**B**) ^NAc^αSyn-Fe^III^ PICUP experiments of ^NAc^WT (grey), ^NAc^Y39F (red), ^NAc^Y125F (yellow), ^NAc^Y133F (green), and ^NAc^Y136F (blue). Bar graphs represent the integrated area PICUP dityrosine signals for (**C**) ^NAc^αSyn-Fe^II^ and (**D**) ^NAc^αSyn-Fe^III^ PICUP experiments.

We then compared the photoinitiated dityrosine crosslinking events in each of the tyrosine mutants following supplementation with either Fe^II^ or Fe^III^ in order to map the crosslinking participation of individual tyrosine residues within the Fe-bound ^NAc^αSyn (Figure 4). Our results further highlight that for ^NAc^αSyn-Fe^II^, each of the tyrosine residues participates in dityrosine crosslinking. There are similar contributions from all of the C-terminal residues of ^NAc^αSyn-Fe^II^, and the predominant contribution results from Y39. The role of Y39 in dityrosine crosslinking within ^NAc^αSyn-Fe^III^ is even more dramatic, with this residue clearly bearing responsibility for the vast majority of the ^NAc^αSyn-Fe^III^ PICUP response. With ^NAc^αSyn-Fe^III^, there is also more of a distinction among the C-terminal tyrosine residues, with Y133 contributing the least to dityrosine crosslinking events since removal of this residue had a less dramatic effect on dityrosine crosslinking among the other ^NAc^αSyn variants.

Collectively, our results suggest that when Fe^III^ binds to ^NAc^αSyn, the tyrosine residues have preferential involvement in overall photochemical crosslinking as follows: Y39 > Y125 > Y136 > Y133 (Figure 4B). In the case of Fe^II^ binding to ^NAc^αSyn, the involvement of tyrosines in crosslinking is as follows: Y39 > Y125 > Y133 > Y136 (Figure 4A). Taken together, these results suggest that when there are changes to the iron redox state, that there is also a change in the coordination environment that results in stabilization of a slightly different ^NAc^αSyn conformation, and thus has a downstream effect on protein crosslinking events. Although the tyrosyl residues are not expected to be involved in Fe coordination, they may be involved in key hydrogen bonding and/or π-stacking stabilization.

### 3.3. Gel Analysis of PICUP Reaction Products

**Figure 5:**
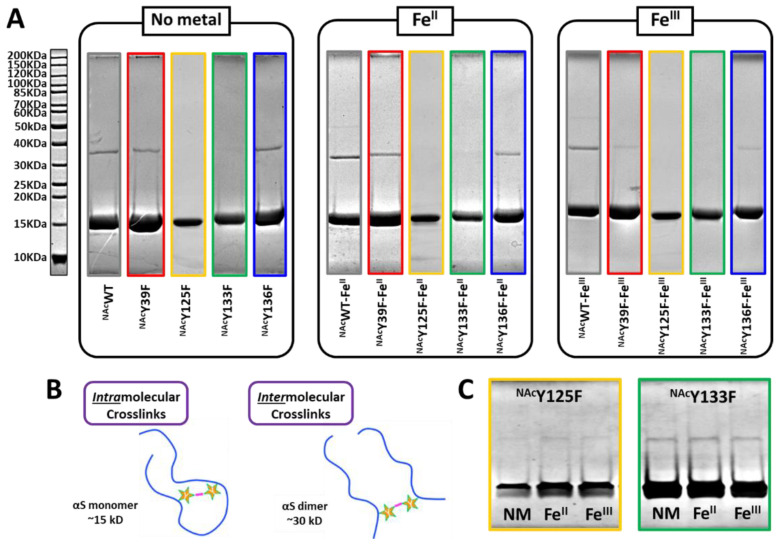
SDS-PAGE analysis of PICUP Samples. (**A**) Samples from PICUP reactions with ^NAc^αSyn variants supplemented with no-metal, Fe^II^_,_ or Fe^III^ separated on 10% SDS-PAGE gels and stained with Coomassie blue R-250. Ladder is located to the left of the gels. (**B**) Schematic representation of intramolecular and intermolecular dityrosine crosslinks. (**C**) Concentrated samples of PICUP reactions performed with ^NAc^Y125F (yellow) and ^NAc^Y133F (green) separated on 10% SDS-PAGE gels stained with Coomassie blue R-250.

In order to correlate the fluorescence emission data with the composition of multimeric crosslinks, PICUP samples were separated on 10% SDS-PAGE gels (Figure 5A). The crosslinking of tyrosine residues takes place when two residues are in close proximity, whether within the fold of a single protein or between two or more proteins appropriately aligned (Figure 5B). Previously, we reported that iron coordination to ^NAc^αSyn does not result in dityrosine crosslinking when exposed to extended aggregation conditions [21]; however, exposure of ^NAc^αSyn as well as ^NAc^αSyn-Fe^III/II^ to blue light under PICUP conditions results in photoinitiated intermolecular dityrosine crosslinks [23]. Based on the results presented herein, Y125 and Y133 are the predominant residues involved in intermolecular crosslinking both in the presence and absence of iron based on the scarcity of a higher molecular weight dimer band within the SDS-PAGE gel of ^NAc^Y125F and ^NAc^Y133F in which these residues have been substituted. In corroboration with our aforementioned fluorescence analyses, Y125 derived crosslinks are the most prevalent since a more dramatic quantum yield decrease results upon its removal. This is also the reason why a relatively higher population of dimers are observed in the ^NAc^Y39F and ^NAc^Y136F samples since both Y125 and Y133 remain intact, and this may further suggest their co-involvement in the crosslink. To confirm the existence of intermolecular crosslinks in ^NAc^Y125F and ^NAc^Y133F since their presence was almost indistinguishable under the conditions used to analyze all protein variants, ^NAc^Y125F and ^NAc^Y133F were concentrated 5-fold are reanalyzed. Indeed, our results verify that intermolecular crosslinks are present within ^NAc^Y125F and ^NAc^Y133F samples and pinpoint Y125 as the key residue involved in these protein coupling events. Our results also demonstrate that neither the Y39 nor Y136 residues are likely to be involved in intermolecular crosslinking since the dimer bands are still present upon their removal and substitution with Phe within the native sequence.

## 4. Conclusions

IDPs like ^NAc^αSyn are often described as natively unfolded, but can actually adopt multiple conformations or partially folded states as part of their intrinsic dynamics or in response to external influences, providing an opportunity for a variety of “native” structural conformations, each with its own potential biological significance. PD is characterized by the abnormal cerebral accumulation of iron, and we have previously demonstrated that the coordination of iron to ^NAc^αSyn influences its self-assembly pathways. In this work, we use the photochemical crosslinking technique PICUP to map the global structural dynamics of ^NAc^αSyn and ^NAc^αSyn-Fe^III/II^ by tracking the individual tyrosine residues that are involved in photoinitiated dityrosine crosslinks. In doing so, we have demonstrated that Y125 is the main contributor to photoinduced dityrosine crosslinks in unmetalated ^NAc^αSyn. We have also established that Fe^III/II^ binding effectively diminishes dityrosine crosslinks in comparison to the unmetalated ^NAc^WT protein. Unlike our previous report in which copper binding promotes intermolecular crosslinking that occurs through a Y39-Y39 linkage, our PICUP results suggest that when Fe binds to the protein, intermolecular crosslinking is favored through participation of C-terminal tyrosine residues, particularly Y125. These studies are particularly relevant to furthering our understanding of the disease state, as the aggregates of ^NAc^αSyn that accumulate within the brain tissue of PD victims are known to contain dityrosine crosslinks. Our results demonstrate that metal ions can alter the folding patterns of natively disordered proteins, which could have an effect on biological pathways as well as protein partner interactions. Moreover, these results shed light on the intrinsic conformational dynamics of ^NAc^αSyn as well as the influence of Fe^III/II^ coordination on the structural configurations accessible to this flexible neuronal protein.
